# 诱导化疗序贯异基因造血干细胞移植治疗FLT3-ITD突变阳性伴正常染色体核型急性髓系白血病的临床研究

**DOI:** 10.3760/cma.j.issn.0253-2727.2023.03.009

**Published:** 2023-03

**Authors:** 芳 李, 燕平 刘, 晗 朱, 鸣 洪, 思轩 钱, 雨 朱, 文怡 沈, 丽娟 陈, 广胜 何, 汉新 吴, 化 陆, 建勇 李, 扣荣 缪

**Affiliations:** 南京医科大学第一附属医院，江苏省人民医院血液科，南京 210029 Department of Hematology, The First Affiliated Hospital of Nanjing Medical University, Nanjing 210029, China

**Keywords:** 白血病，髓系，急性, FLT3-ITD突变, 异基因造血干细胞移植, 预后, Leukemia, myeloid, acute, FLT3-ITD mutation, Allogeneic hematopoietic stem cell transplantation, Prognosis

## Abstract

**目的:**

评估诱导化疗序贯异基因造血干细胞移植治疗FLT3-ITD突变阳性伴正常染色体核型急性髓系白血病（AML）的疗效。

**方法:**

对2018年1月至2021年3月南京医科大学第一附属医院收治的FLT3-ITD^+^伴正常染色体核型AML患者进行回顾性分析。

**结果:**

49例FLT3-ITD^+^AML患者纳入研究，男31例，女18例，中位年龄46（16～59）岁。所有患者均接受诱导化疗，24例患者序贯异基因造血干细胞移植（移植组）。中位随访时间为465 d，确诊后1年总生存（OS）率为（70.0±7.4）％，1年无病生存（DFS）率为（70.3±7.4）％。移植组、非移植组1年OS率分别为（85.2±7.9）％、（52.6±12.3）％（*P*＝0.049），DFS率分别为（84.7±8.1）％、（55.2±11.9）％（*P*＝0.061）。在移植组和非移植组中，FLT3-ITD低频突变与高频突变患者1年OS率差异均无统计学意义（*P*>0.05）。移植组、非移植组中各有12例FLT3-ITD高频突变患者，1年OS率分别为（68.8±15.7）％、（26.2±15.3）％（*P*＝0.027），1年DFS率分别为（45.5±21.3）％、（27.8±15.8）％（*P*＝0.032）。

**结论:**

诱导化疗序贯allo-HSCT可改善FLT3-ITD^+^患者（特别是FLT3-ITD高频突变患者）的预后。

## Abstract (en)

**Objective:**

To assess the efficacy of induction chemotherapy followed by allogeneic hematopoietic stem cell transplantation（allo-HSCT）in the treatment of FLT3-ITD^+^ acute myeloid leukemia（AML）with normal karyotype.

**Methods:**

The clinical data of FLT3-ITD^+^ AML patients with normal karyotype in the First Affiliated Hospital of Nanjing Medical University from Jan 2018 to March 2021 were retrospectively analyzed.

**Results:**

The study included 49 patients with FLT3-ITD^+^AML, 31 males, and 18 females, with a median age of 46（16–59）years old. All patients received induction chemotherapy, and 24 patients received sequential allo-HSCT（transplantation group）. The median follow-up time was 465 days, the one-year overall survival（OS）from diagnosis was（70.0 ± 7.4）％, and one-year disease-free survival（DFS）was（70.3±7.4）％. The one-year OS was significantly different between the transplantation group and the non-transplantation group［（85.2 ± 7.9）％ *vs*（52.6 ± 12.3）％, *P*＝0.049］. but one-year DFS［（84.7 ± 8.1）％ *vs*（55.2 ± 11.9）％, *P*＝0.061］was not. No significance was found in one-year OS between patients with low-frequency and high-frequency FLT3-ITD^+^（*P*>0.05）. There were 12 patients with high-frequency FLT3-ITD^+^ in the transplantation and the non-transplantation groups, respectively. The one-year OS［（68.8 ± 15.7）％ in the transplantation group *vs*（26.2 ± 15.3）％ in the non-transplantation group, *P*＝0.027］and one-year DFS［（45.5 ± 21.3）％ in the transplantation group *vs*（27.8±15.8）％ in the non-transplantation group, *P*＝0.032］were significantly different between the two groups.

**Conclusion:**

Induction chemotherapy followed by allo-HSCT can enhance the prognosis of FLT3-ITD^+^ patients, particularly those with FLT3-ITD high-frequency mutation.

急性髓系白血病（AML）是造血系统异常增殖、克隆分化的一种恶性肿瘤，其细胞遗传学改变及克隆演变多种多样[1]。FMS样酪氨酸激酶3（FLT3）基因定位于13号染色体长臂（13q12），FLT3突变在成人AML中的检出率为25％～30％[2]，其中FLT3-ITD检出率为19％～25％[3]。FLT3-ITD^+^可持续激活FLT3，导致多种下游通路异常激活，引起造血干细胞异常增殖、凋亡受抑和分化受阻，导致白血病的发生和进展[4]，临床预后不良[5]。2017年欧洲白血病工作网（ELN）推荐使用FLT3-ITD等位基因比值（AR）及有无NPM1突变对FLT3-ITD^+^ AML患者进行危险分层，但美国综合癌症中心（NCCN）指南仍然推荐所有FLT3-ITD^+^AML患者行异基因造血干细胞移植（allo-HSCT），无论其AR值或NPM1突变状态如何[6]。在核型正常且疾病处于完全缓解（CR）状态下，FLT3-ITD^+^ AML患者是否能通过诱导化疗序贯allo-HSCT进一步改善预后尚无定论。本研究纳入49例年龄16～60岁、染色体核型正常FLT3-ITD^+^AML患者，评估诱导化疗序贯allo-HSCT的疗效并分析高频FLT3-ITD突变的预后意义。

## 病例与方法

一、病例

本回顾性研究纳入2018年1月至2021年3月于南京医科大学第一附属医院（江苏省人民医院）血液科接受诊治的49例FLT3-ITD突变阳性伴正常染色体核型AML患者。所有患者根据细胞形态学、免疫学、细胞遗传学、分子生物学（MICM）分型标准确诊，采用二代测序（NGS）方法进行基因检测，并计算FLT3-ITD的AR值。留取骨髓液经R显带法分析染色体核型，根据《人类细胞遗传学国际命名体制（ISCN2013）》进行描述。

二、诱导化疗

所有49例患者在诊断后均接受常规剂量的IA（去甲氧柔红霉素+阿糖胞苷）、DA（柔红霉素+阿糖胞苷）或地西他滨+CAG（阿克拉霉素+阿糖胞苷+ G-CSF）方案进行诱导治疗，获得CR后行3～4次中大剂量阿糖胞苷为基础的标准巩固化疗。部分患者化疗过程中联合服用索拉非尼。

三、移植方案

有移植意愿且有合适供者的患者在诱导化疗后序贯allo-HSCT。移植组24例患者中，19例行单倍体造血干细胞移植（haplo-HSCT），5例接受亲缘同胞全相合供者干细胞移植。预处理方案：基于白消安（Bu）/环磷酰胺（Cy）方案17例，氟达拉滨（Flu）/白消安（Bu）方案7例。移植物均为外周血造血干细胞，单个核细胞（MNC）中位输注量为6.86（4.59～17.81）×10^8^/kg，CD34^+^细胞中位输注量为5.02（2.12～19.13）×10^6^/kg。移植物抗宿主病（GVHD）预防采用环孢素A（CsA）联合短程甲氨蝶呤，无关供者移植及haplo-HSCT患者加用兔抗人胸腺细胞免疫球蛋白（rATG）及霉酚酸酯（MMF），当发生Ⅱ～Ⅳ级急性GVHD时，采用糖皮质激素作为一线治疗方案。

四、随访

随访时间截至2021年11月10日，采用门诊、住院检查及电话等方式进行随访，常规监测血常规、生化、病毒及骨髓象。根据国际标准[7]定义CR和复发。总生存（OS）期定义为初诊至患者死亡、末次随访或失访的时间，无病生存（DFS）期定义为初诊至复发、死亡或末次随访的时间。中性粒细胞植入定义为连续3 d中性粒细胞计数>0.5×10^9^/L，血小板植入定义为连续7 d血小板计数>20×10^9^/L且脱离血小板输注。采用PCR方法检测巨细胞病毒（CMV）及EB病毒（EBV），连续两次CMV-DNA>1000拷贝数/ml为CMV血症，连续两次EBV-DNA>1000拷贝数/ml为EBV血症，急性GVHD和慢性GVHD的诊断和分级按照文献[8]。

五、统计学处理

OS和DFS采用Kaplan-Meier概率法计算，非复发死亡（NRM）采用竞争风险模型（Grey检验）来计算，*P*<0.05为差异有统计学意义。分类变量比较采用卡方检验，连续变量比较采用非参数Mann-Whitney *U*检验确定中位值差异。所有数据分析使用Graphpad 8、SPSS 23软件包完成。

## 结果

一、患者一般临床资料

49例FLT3-ITD阳性AML患者中男31例，女18例，中位年龄46（16～59）岁；非移植组25例，男15例，女10例，中位年龄50（28～59）岁；移植组24例，男16例，女8例，中位年龄43（16～53）岁。移植组中位年龄低于非移植组（*P*＝0.012），其余临床特征差异无统计学意义（表1）。

**表1:** 49例FLT3-ITD阳性伴正常染色体核型急性髓系白血病（AML）患者基本临床资料

基本信息	移植组（24例）	非移植组（25例）	统计量	*P值*
性别（例）			*χ*^2^=0.234	0.628
男	16	15		
女	8	10		
年龄［岁，*M*（范围）］	43（16~53）	50（28~59）	*z*=2.502	0.012
初诊WBC［×10^9^/L，*M*（范围）］	29.6（0.8~246.7）	35.8（1.8~561.0）	*z*=0.480	0.631
初诊PLT［×10^9^/L，*M*（范围）］	55（8~542）	63（9~659）	z=0.325	0.884
初诊HGB［g/L，*M*（范围）］	88（37~142）	84（41~142）	*z*=0.340	0.734
初诊乳酸脱氢酶［U/L，*M*（范围）］	376.5（124~1 172）	467（140~3 995）	*z*=1.360	0.174
分型（例）			*χ*^2^=0.717	0.869
M_1_	10	8		
M_2_	10	12		
M_4_	1	2		
M_5_	3	3		
骨髓原始细胞［％，*M*（范围）］	66.4（22.2~96.0）	76.0（35.0~96.0）	*z*=1.821	0.069
高频FLT3-ITD突变（例）	12	12	*χ*^2^=0.002	0.889
诱导化疗疗程>1（例）	7	9	*χ*^2^=0.260	0.610
初次诱导化疗获得CR（例）	16	15	*χ*^2^=0.234	0.628
联合服用索拉非尼（例）	9	5	*χ*^2^=1.838	0.175
随访时间［d，*M*（范围）］	466.5（170~1 643）	420（73~1 352）	*z*=1.932	0.135

**注** M_1_：急性髓系白血病未分化型；M_2_：急性髓系白血病部分分化型；M_4_：急性粒-单核细胞白血病；M_5_：急性单核细胞白血病；CR：完全缓解；高频FLT3-ITD突变：等位基因比值≥0.5

二、FLT3-ITD阳性患者伴随基因突变情况

49例患者中位伴随基因突变数为3（1～6）种，共涉及21种基因，突变率较高的前5种基因分别为TET2（51.0％）、NPM1（30.6％）、WT1（28.6％）、DNMT3A（24.5％）和NRAS（20.4％）。DNA甲基化相关基因突变如TET2及DNMT3A突变在移植组和非移植组中都很常见。两组在伴随基因突变分布上差异无统计学意义（*P*>0.05）（表2）。

**表2:** 移植组和非移植组FLT3-ITD阳性伴正常染色体核型急性髓系白血病患者伴随基因突变分布（例）

突变类型	移植组（24例）	非移植组（25例）	*χ*^2^值	*P*值
TET2突变			0.186	0.666
有	13	12		
无	11	13		
DNMT3A突变			0.556	0.456
有	7	5		
无	17	20		
WT1突变			0.523	0.47
有	8	6		
无	16	19		
NPM1突变			0.046	0.83
有	7	8		
无	17	17		
NRAS突变			0.405	0.524
有	4	6		
无	20	19		
RUNX1突变			0.003	0.957
有	3	3		
无	21	22		
C-kit突变			0.002	0.966
有	2	2		
无	22	23		

三、治疗反应

非移植组25例患者中15例（60.0％）于初次诱导后获得CR，5例（20.0％）复发，9例（36.0％）死亡（5例死于肺部感染，4例死于感染性休克）。移植组患者移植前疾病状态：第1次CR（CR_1_）期15例（62.5％），≥CR_2_期9例（37.5％）。移植后4例复发，7例（29.2％）死亡（3例死于肺部感染，2例死于多脏器功能衰竭，2例死于感染性休克）。所有移植患者均获得中性粒细胞及血小板植入，中性粒细胞植入的中位时间为13（10～19）d，血小板植入的中位时间为13（8～22）d。移植后100 d急性GVHD发生率为36.3％（8例），Ⅱ～Ⅳ级急性GVHD发生率为27.3％（6例），移植后1年慢性GVHD发生率为14.3％（3例）。CMV血症16例，中位发生时间为移植后36（20～45）d；EBV血症15例，中位发生时间为移植后56（34～82）d，未发生淋巴细胞增殖性疾病。

四、生存分析

中位随访时间为465（73～1 643）d。至随访截止，34例患者存活，15例死亡；1年OS率为（70.0±7.4）％，1年DFS率为（70.3±7.4）％。移植组1年OS率高于非移植组［（85.2±7.9）％对（52.6±12.3）％，*P*＝0.049］，1年DFS率差异无统计学意义［（84.7±8.1）％对（55.2±11.9）％，*P*＝0.061］，生存曲线见图1。

**图1:**
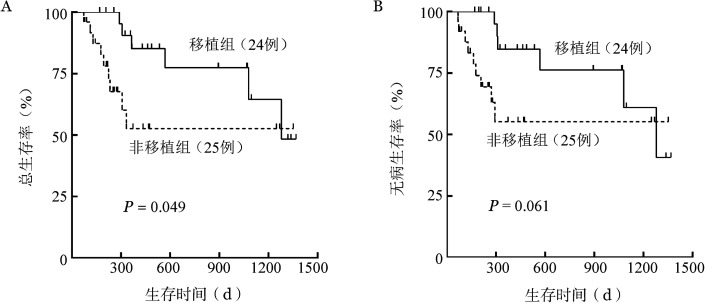
移植组和非移植组FLT3-ITD突变阳性伴正常染色体核型急性髓系白血病患者总生存曲线（A）和无病生存曲线（B）

五、FLT3-ITD高频突变、低频突变AML患者预后的比较

非移植组中，FLT3-ITD高频突变（FLT3-ITD^+^ AR≥0.5）、低频突变（FLT3-ITD^+^ AR<0.5）患者1年OS率分别为（26.2±15.3）％、（81.8±11.6）％（*P*＝0.064）（图2A）。移植组中，FLT3-ITD高频突变、低频突变患者1年OS率分别为（82.5±11.3）％、（88.9±10.5）％（*P*＝0.151）（图2B）。

**图2:**
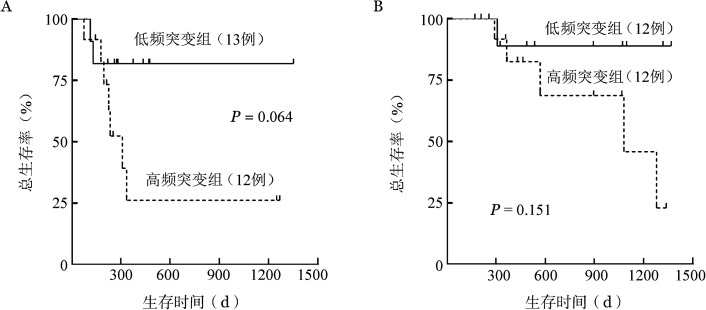
FLT3-ITD高频突变对非移植组（A）和移植组（B）伴正常染色体核型急性髓系白血病患者总生存期的影响

六、移植组和非移植组FLT3-ITD高频突变患者预后的比较

移植组、非移植组中各有12例FLT3-ITD高频突变患者，1年OS率分别为（68.8±15.7）％、（26.2±15.3）％（*P*＝0.027），1年DFS率分别为（45.5±21.3）％、（27.8±15.8）％（*P*＝0.032）。生存曲线见图3。

**图3:**
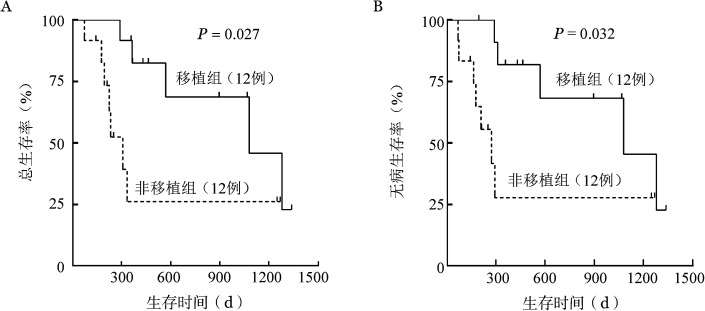
移植和非移植治疗FLT3-ITD高频突变伴正常染色体核型急性髓系白血病患者总生存曲线（A）和无病生存曲线（B）

## 讨论

FLT3-ITD^+^ AML预后不良，建议在CR_1_期进行allo-HSCT[9]，同时高等位基因突变负荷的不良预后可能会被克服[10]；但移植作为FLT3-ITD^+^正常核型AML低、中危患者的治疗仍有争议[11]。

FLT3-ITD^+^ AML患者是否适合allo-HSCT取决于FLT3-ITD突变负荷、伴随突变、化疗获得CR后微小残留病（MRD）及FLT3抑制剂使用等情况[12]–[13]。FLT3-ITD^+^ AR值对生存结果有着重要影响，尽管不同研究中心对于AR的截断值定义不同，但0.5是2017年ELN指南基于大量临床研究结果作出的推荐[14]。一项评估FLT3突变AML患者预后因素的研究结果显示，FLT3-ITD^+^ AR≥0.78的患者OS和DFS显著缩短，而AR<0.78患者的生存与无FLT3突变患者差异无统计学意义[15]。FLT3-ITD^+^AR值纳入常规临床实践的局限性在于缺乏明确的阈值，ELN建议为0.5，也有研究者将AR截断值设定为0.7[16]。FLT3-ITD AR值相关研究迄今为止更多的是回顾性研究，尚缺乏前瞻性研究[13]。本研究分别在移植组和非移植组中分析FLT3-ITD AR值对生存预后的影响，使用0.5作为AR值的截断值划分高频突变组和低频突变组，并未发现FLT3-ITD高频突变与低频突变两组间差异有统计学意义（*P*>0.05）。接着比较移植和非移植治疗FLT3-ITD高频突变AML患者的OS，发现移植能改善该部分患者的预后，移植组1年OS率及DFS率显著高于非移植组（*P*<0.05）。但对于利用FLT3-ITD^+^ AR指导缓解后治疗及是否立即进行移植仍存在争议。

FLT3-ITD^+^ AML较其他类型AML患者更容易且更早发生复发。在仅接受化疗的细胞遗传学正常AML患者中，与FLT3-ITD突变相关的预后判断取决于AR值和伴随的NPM1突变。指南建议初诊AML进行二代测序检测分子突变，对FLT3突变AML患者早期给予靶向药物以实现更深程度的缓解，早期考虑allo-HSCT[17]。Oran等[18]发现，在FLT3-ITD^+^ AML患者中，获得CR_1_后行allo-HSCT可改善DFS和OS。Sakaguchi等[19]对147例FLT3-ITD/NPM1共突变患者进行了研究，FLT3-ITD低频突变伴NPM1阳性的AML患者常规化疗的OS率仅为41％，但在CR_1_期进行allo-HSCT患者的DFS和OS均有显著改善。赵辉等[20]报道了56例FLT3-ITD^+^ AML移植患者的临床资料，3年OS率、DFS率分别为71.2％、65.6％，提示allo-HSCT可改善FLT3-ITD^+^ AML患者的预后。欧洲血液及骨髓移植协会（EBMT）对于中高危FLT3-ITD^+^ AML患者，建议在CR_1_后行allo-HSCT[14]。本研究主要分析了移植患者和非移植患者的生存情况，结果提示诱导化疗获得CR后序贯allo-HSCT可进一步改善FLT3-ITD高频突变AML患者的OS及DFS。但上述结论尚需进一步验证。

随着FLT3抑制剂新药的出现，诸多前瞻性及回顾性研究均证实在FLT3-ITD^+^ AML的一线治疗中，无论AR值大小，应用移植或化疗联合靶向药物均可使患者生存有所改善[21]。服用索拉菲尼治疗的患者数量较少，这是本研究的最大缺陷。
